# Supplementary material for: Structural basis of pausing during transcription initiation in mycobacterium tuberculosis
Source: Nat Commun. 2026 Jan 30;17:2197. doi: 10.1038/s41467-026-69104-w (PMC12960844; doi:10.1038/s41467-026-69104-w)
Supplement: Supplementary file 1 — Supplementary Information [file 41467_2026_69104_MOESM1_ESM.pdf]

Supplementary Figures

Supplementary Figure 1

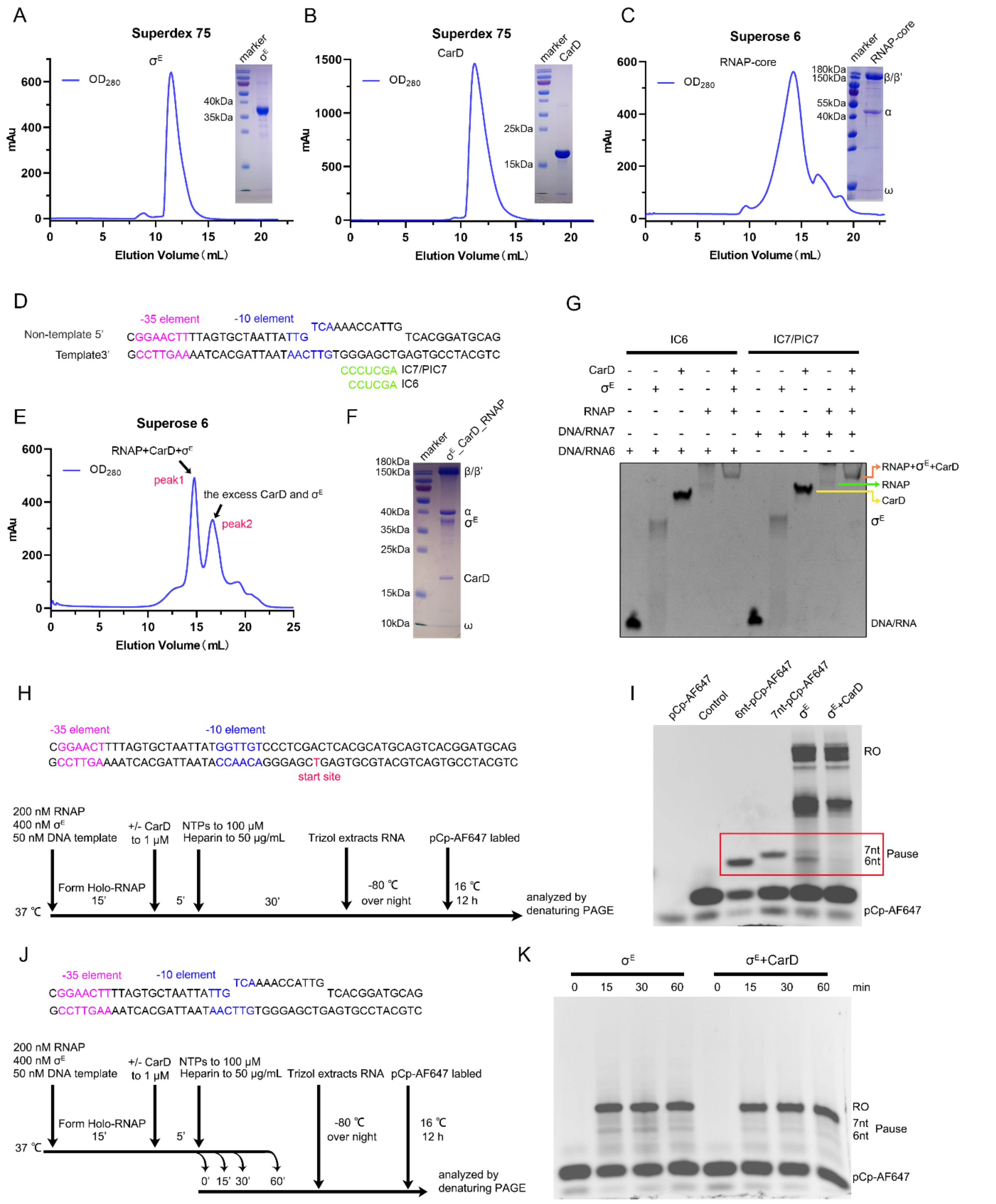

Supplementary Figure 1 | Purification and characterization of σ^E, CarD, RNAP-core, and their complexes (related to Figure 1).

A-C. Size-exclusion chromatography (SEC) elution profiles of purified $σ^E$ **(A)**, CarD **(B)**, and *Mtb* RNAP-core enzyme **(C)**. SDS-PAGE analysis of SEC-purified $σ^E$, CarD, and RNAP; gels were stained with Coomassie blue.

D. The nucleic-acid scaffold used for structure determination.

E. Elution peaks of $σ^E$_CarD_RNAP complex from a size exclusion column. Peak 1 is the $σ^E$_CarD_RNAP complex and peak 2 are the excess $σ^E$ and CarD.

F. SDS-PAGE analysis of the major SEC peak (Peak 1) from **(E)**, confirming co-migration of all three components.

G. EMSA verifying successful assembly of the $σ^E$-CarD-RNAP complex with the scaffold in **(D)**. n = 3 independent experiments. Source data are provided as a Source Data file.

H. Promoter DNA templates used for in vitro transcription assays with $σ^E$ and CarD. The schematic below illustrates the fully matched promoter template architecture corresponding to the reactions shown in **(I)**, including the promoter sequence and the expected RNA products.

I. In vitro transcription assays showing initiation pausing by $σ^E$ and the effect of CarD using the fully matched template. Control reactions lacked promoter DNA. $σ^E$ and $σ^E$+CarD indicate run-off transcription with or without CarD. Synthetic 6-nt and 7-nt RNAs labeled with pCp–AF647 were used as size markers. n = 3 independent experiments. Source data are provided as a Source Data file.

J. Schematic of the in vitro transcription assay using a pre-melted promoter scaffold, analogous to **(H)** but with a pre-opened bubble.

K. In vitro transcription using the pre-melted template. A time course monitors early pausing, and $σ^E$+CarD shows reduced 6–7 nt accumulation, consistent with the fully matched template. n = 3 independent experiments. Source data are provided as a Source Data file.

Supplementary Figure 2

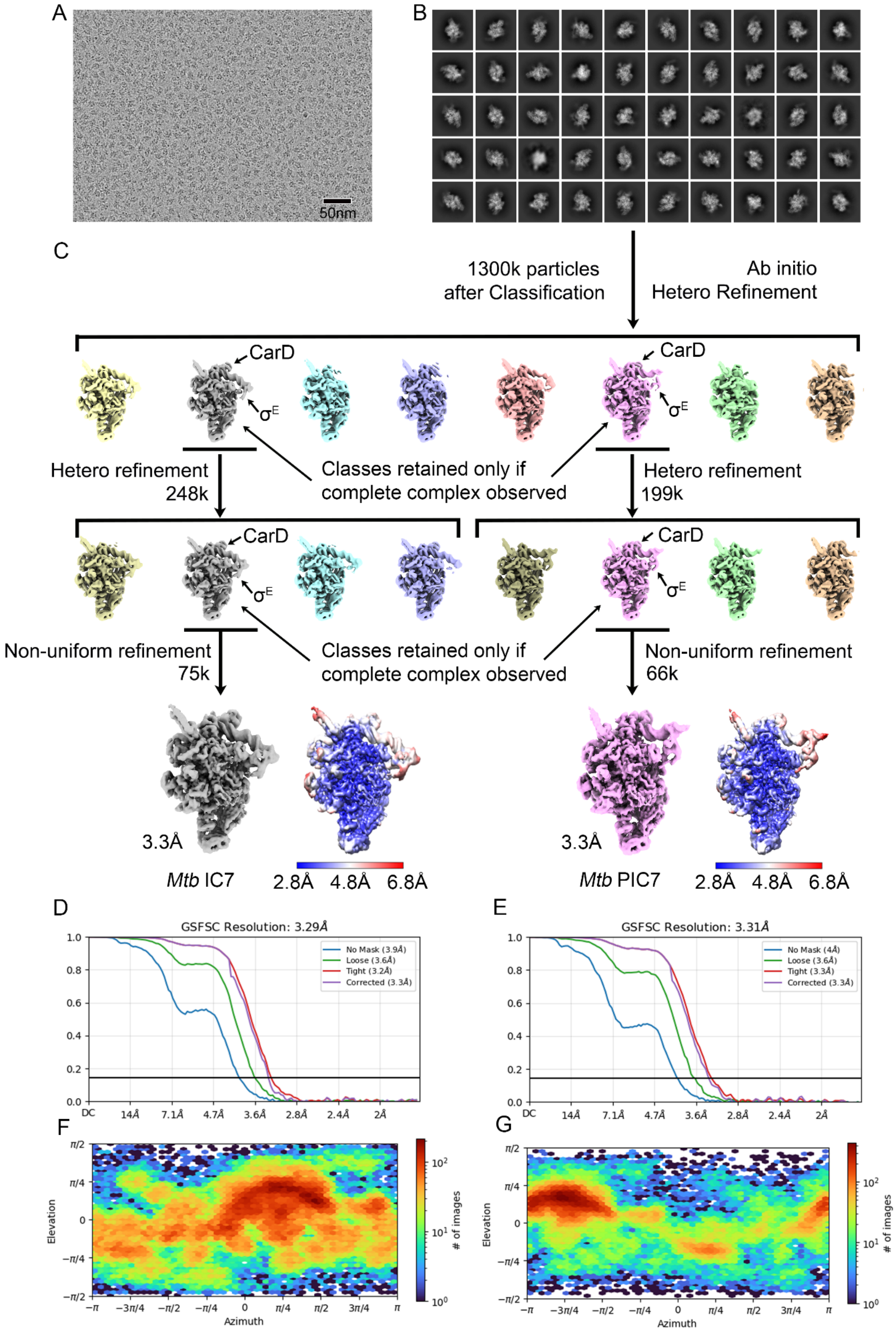

Supplementary Figure 2 | Data processing workflow and Cryo-EM map validation for *Mtb* IC7 and PIC7 (related to Figure 1).

A. Raw micrograph of the *Mtb* IC7 and PIC7 particles in vitreous ice recorded at defocus values of -1.0 to -1.8 μm. Scale bar, 50 nm.

B. Representative 2D class averages. The edge of each square is ~320 Å.

C. Schematic workflow for 3D reconstruction of *Mtb* IC7 and PIC7. Particle classes containing the complete σ^E-CarD-RNAP-DNA/RNA complex were retained for further refinement, while classes lacking one or more components were excluded.The bottom panel displays the local resolution map (color-coded from 2.8 Å (blue) to 6.8 Å (red)). The lower resolution of σ4 and DNA indicates flexibility of this region.

D, E. Gold-standard Fourier Shell Correlation (FSC) curves for the final refined map.

F, G. Angular distribution heatmap of particle orientations.

Supplementary Figure 3

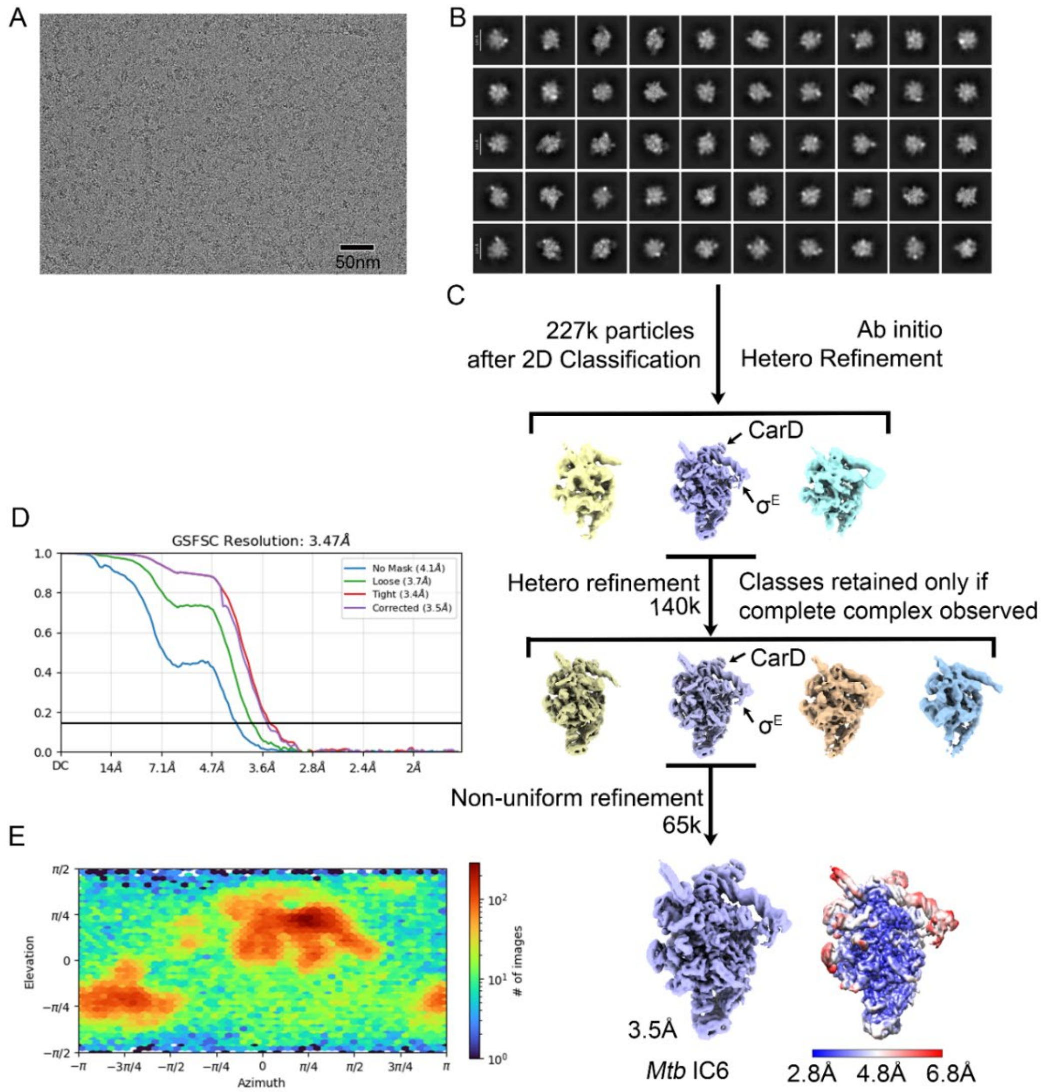

Supplementary Figure 3 | Data processing workflow and Cryo-EM map validation for *Mtb* IC6 (related to Figure 1).

A. Raw micrograph of the *Mtb* IC6 particles in vitreous ice recorded at defocus values of -1.0 to -1.8 μm. Scale bar, 50 nm.

B. Representative 2D class averages. The edge of each square is ~320 Å.

C. Schematic workflow for 3D reconstruction of *Mtb* IC6. Particle classes containing the complete σ^E-CarD-RNAP-DNA/RNA complex were retained for further refinement, while classes lacking one or more components were excluded. The bottom panel displays the local resolution map (color-coded from 2.8 Å (blue) to 6.8 Å (red)). The lower resolution of σ4 and DNA indicates flexibility of this region.

D. Gold-standard Fourier Shell Correlation (FSC) curves for the final refined map.

E. Angular distribution heatmap of particle orientations.

Supplementary Figure 4

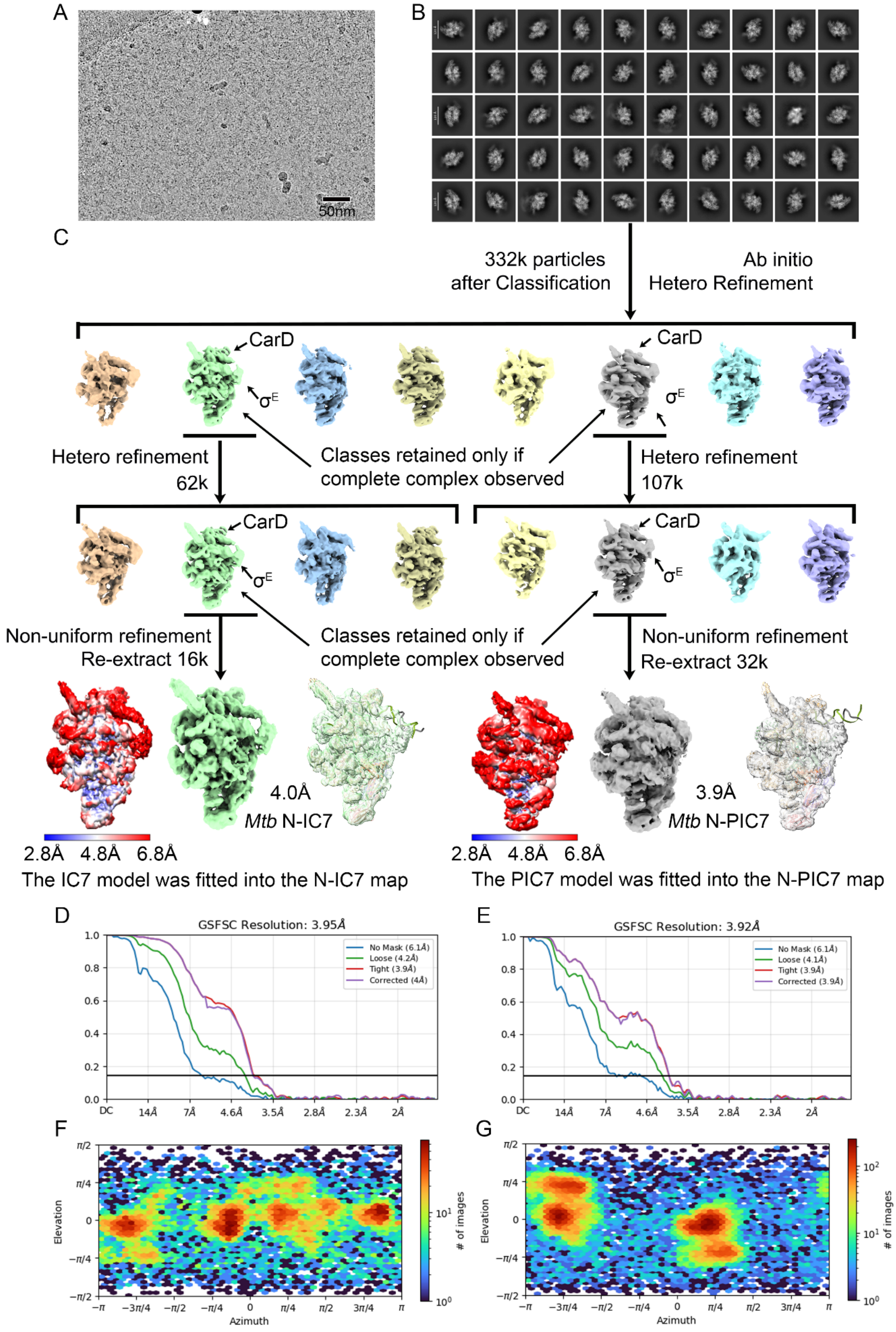

Supplementary Figure 4 |Data processing workflow and Cryo-EM map validation for *Mtb* N-IC7 and N-PIC7 (related to Figure 1).

A. Raw micrograph of the *Mtb* N-IC7 and N-PIC7 particles in vitreous ice recorded at defocus values of -1.0 to -1.8 µm. Scale bar, 50 nm.

B. Representative 2D class averages. The edge of each square is ~320 Å.

C. Schematic workflow for 3D reconstruction of *Mtb* N-IC7 and N-PIC7. Particle classes containing the complete σ^E-CarD-RNAP-DNA/RNA complex were retained for further refinement, while classes lacking one or more components were excluded. The bottom panel displays the models derived from IC7 and PIC7 fitted into the maps of N-IC7 and N-PIC7, respectively.

D, E. Gold-standard Fourier Shell Correlation (FSC) curves for the final refined map.

F, G. Angular distribution heatmap of particle orientations.

Supplementary Figure 5

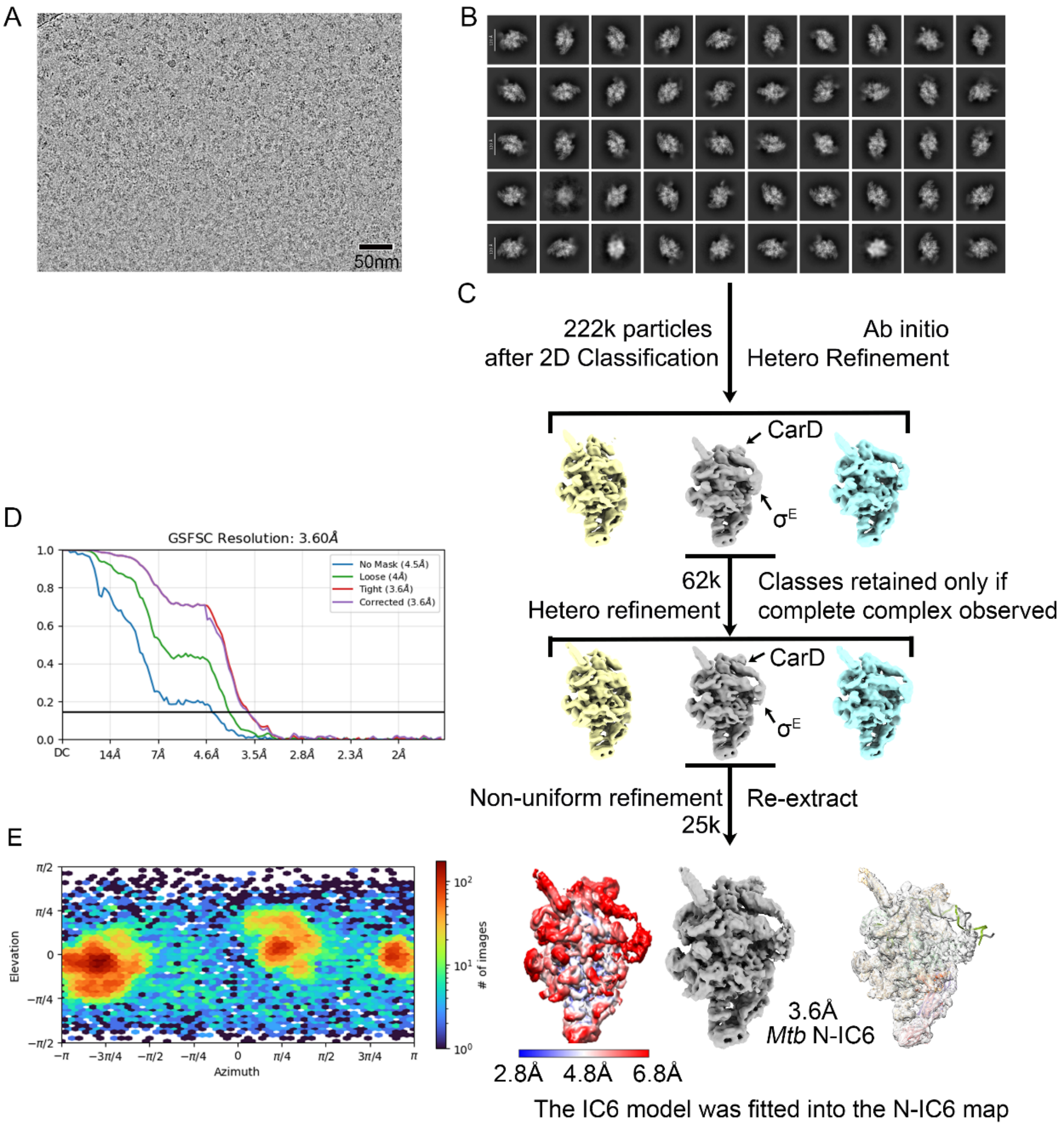

Supplementary Figure 5 | Data processing workflow and Cryo-EM map validation for *Mtb* N-IC6 (related to Figure 1).

A. Raw micrograph of the *Mtb* N-IC6 particles in vitreous ice recorded at defocus values of -1.0 to -1.8 µm. Scale bar, 50 nm.

B. Representative 2D class averages. The edge of each square is ~320 Å.

C. Schematic workflow for 3D reconstruction of *Mtb* N-IC6. Particle classes containing the complete σ^E-CarD-RNAP-DNA/RNA complex were retained for further refinement, while classes lacking one or more components were excluded. The bottom panel displays the model obtained from IC6 was fitted into the map of N-IC6.

D. Gold-standard Fourier Shell Correlation (FSC) curves for the final refined map.

E. Angular distribution heatmap of particle orientations.

Supplementary Figure 6

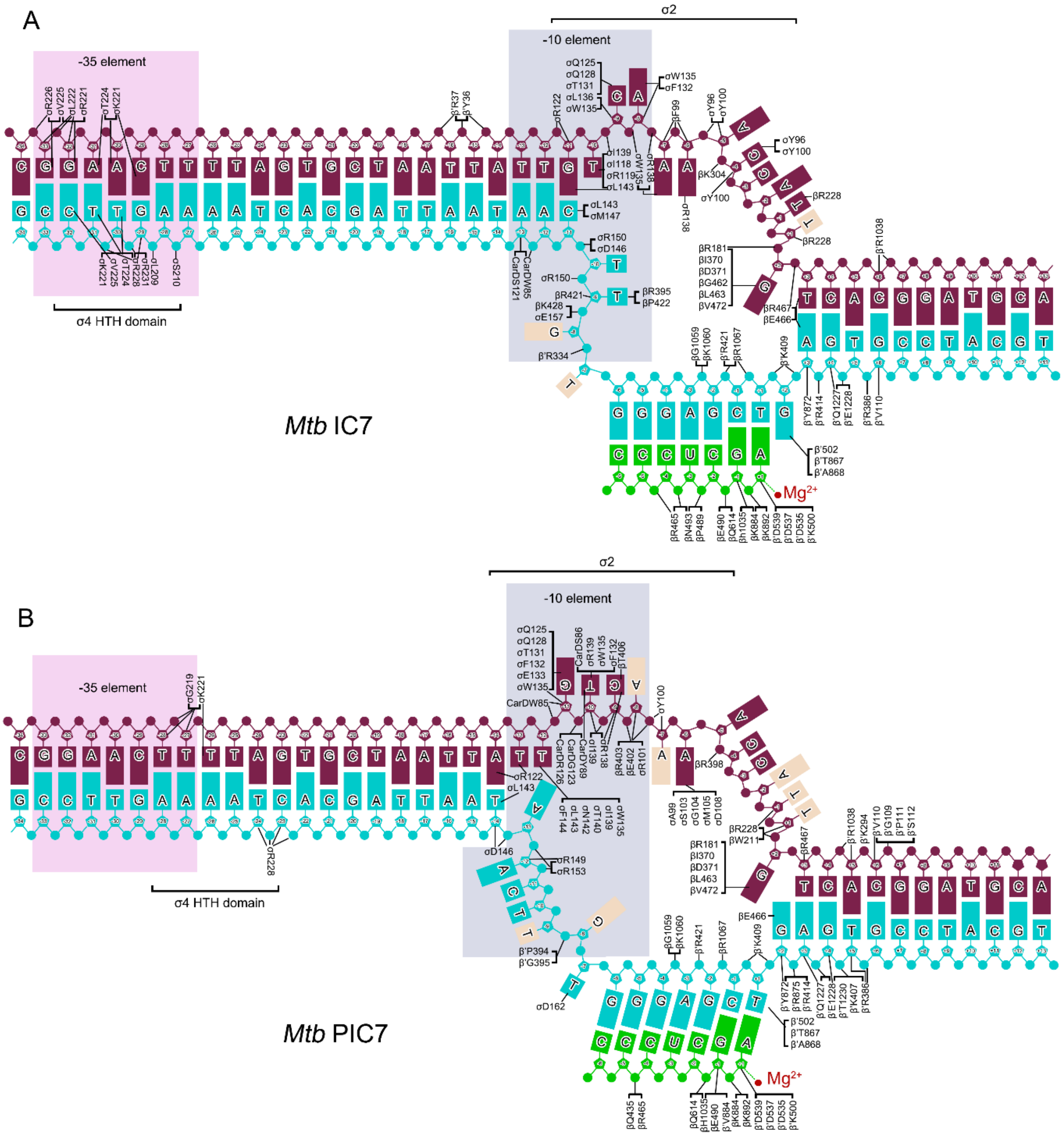

Supplementary Figure 6 | Structural characterization of protein-DNA/RNA interactions in IC7 and PIC7 (related to Figure 1).

A, B. Summary of protein-DNA/RNA interactions in IC7 **(A)** and PIC7 **(B)**. The color scheme for template, non-template, and RNA are in cyan, warm pink, and green, respectively. Flexible nucleotide bases are highlighted in wheat. Pink boxes denote interactions with the −35 promoter element; light blue boxes indicate contacts with the −10 element.

Supplementary Figure 7

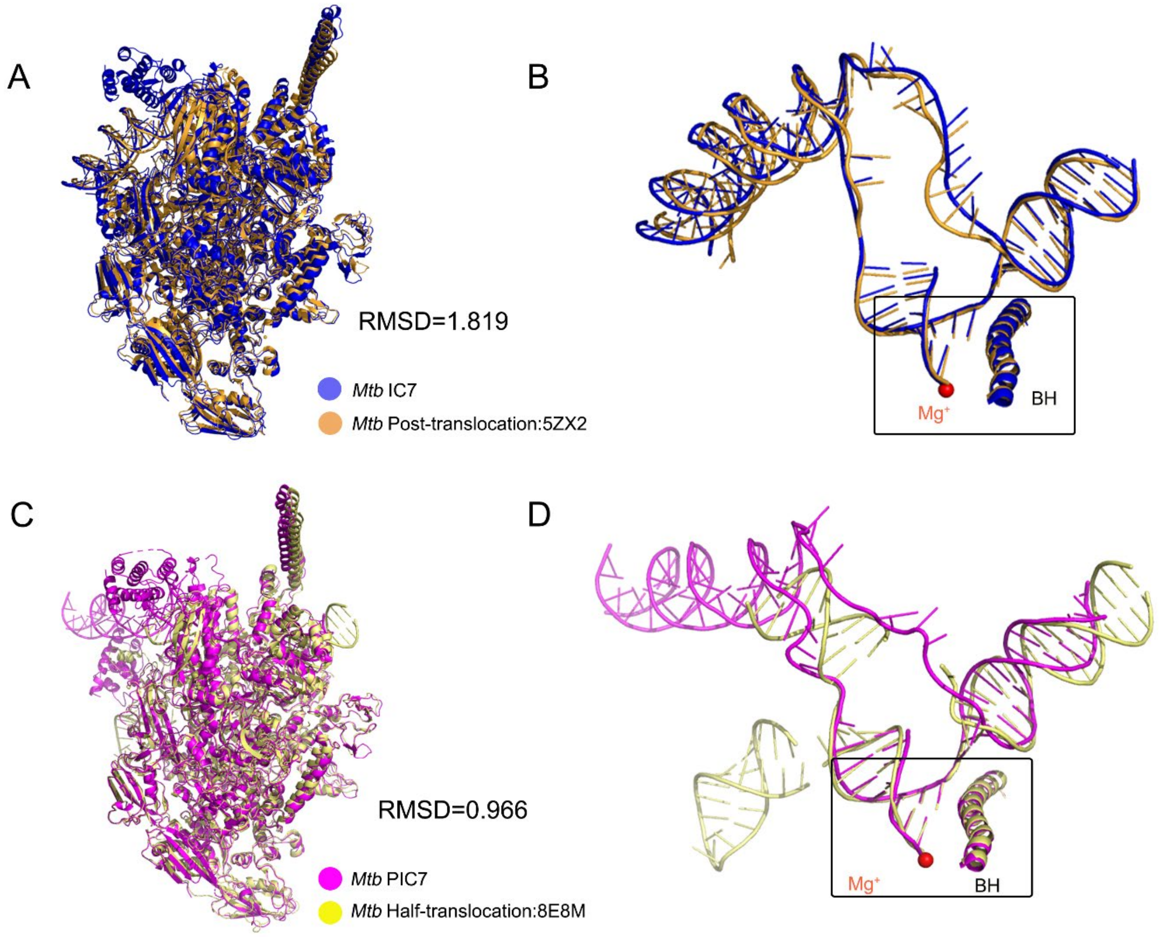

Supplementary Figure 7 | Structural comparisons of transcription initiation complexes (related to Figure 1, 2).

A. Structural alignment of *Mtb* IC7 (blue) and *Mtb* σ^H-RPo (orange; PDB: 5ZX2) reveals conserved RNAP-core conformations in the post-translocation state.

B. The comparison of upstream double-stranded DNA (dsDNA), transcription bubble, and downstream dsDNA in IC7 and *Mtb* σ^H-RPo.

C. Structural alignment of *Mtb* PIC7 (magenta) and *Mtb* PEC (yellow; PDB: 8E8M) reveals conserved RNAP-core conformations in the half-translocation state.

D. The comparison of upstream double-stranded DNA (dsDNA), transcription bubble, and downstream dsDNA in PIC7 and *Mtb* PEC.

Supplementary Figure 8

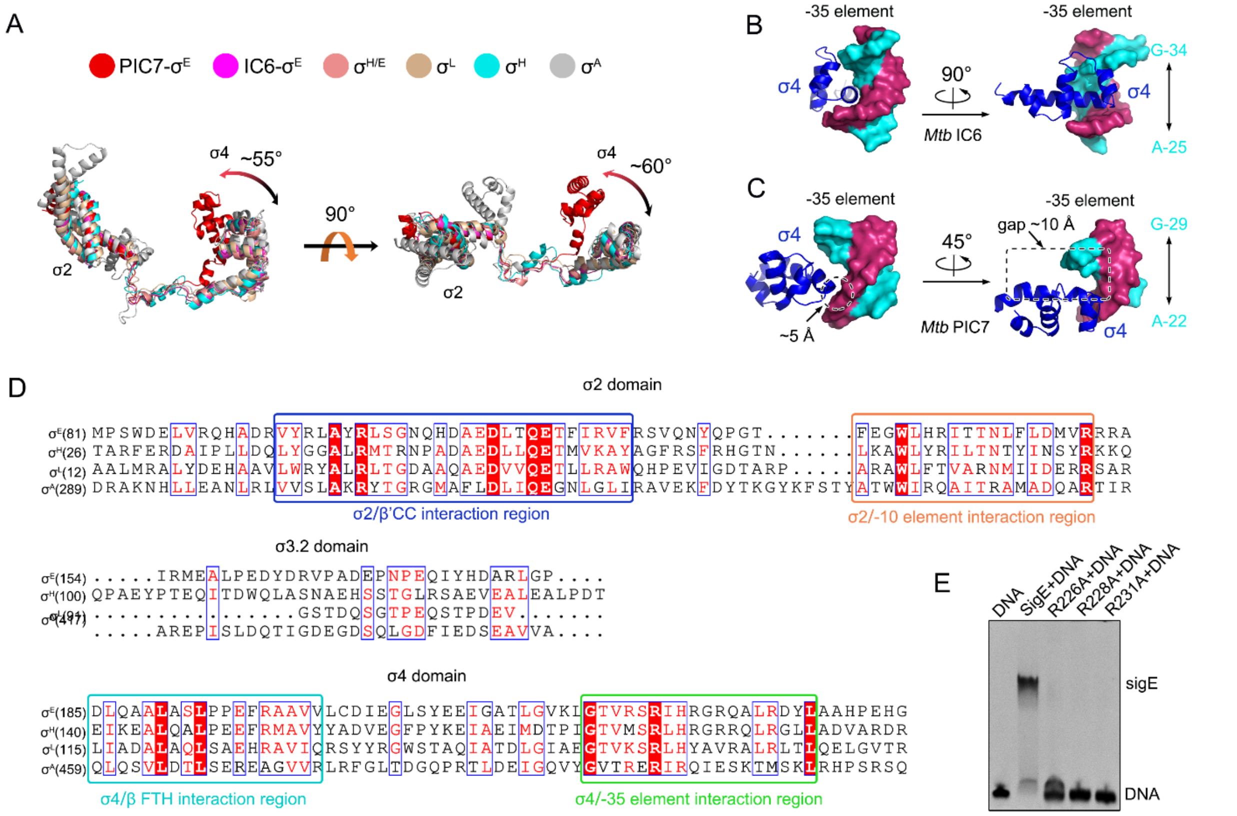

Supplementary Figure 8 | Comparison of transcription initiation factor (related to Figure 2, 3).

A. Structural superposition of σ factor homologs aligned through their σ4 domains, including σ^H (PDB: 5ZX2; cyan), σ^A (PDB: 5UH5; gray), σ^L (PDB: 6DVD; wheat), $\sigma^{H/E}$ variant (PDB: 6JCY; salmon), σ^E-IC6 (PDB: 9M98; magenta), and σ^E-PIC7 (PDB: 9M9E; red). All structures are shown in identical orientations to emphasize spatial conservation of the σ4 domain.

B, C. σ4 domain engagement with promoter -35 element. IC6 **(B)** and PIC7 **(C)** structures shown in dual orientations, which also show different modes of combination.

D. Sequence alignment of σ factors identifies conserved motifs for promoter recognition and RNAP interaction: Residues contacting the -10 element (salmon boxes) and -35 element (green boxes) define promoter-binding regions in σ2 and σ4 domains, respectively; regions mediating RNAP interactions are marked by blue (σ2-RNAP interface) and cyan (σ4-RNAP interface) boxes.

E. EMSA quantifies the effect of σ4 on DNA binding affinity using DNA alone (the DNA substrate shown in Supplementary Figure 1H). n = 3 independent experiments. Source data are provided as a Source Data file.

Supplementary Figure 9

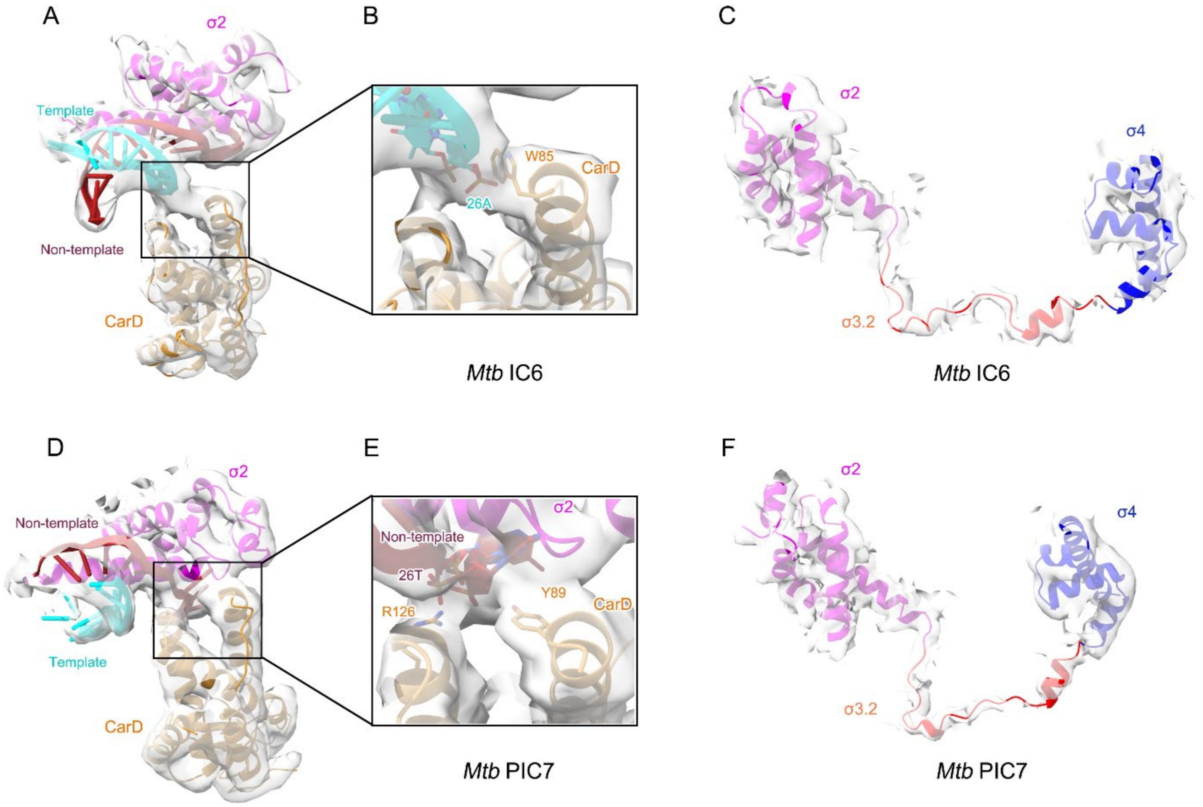

Supplementary Figure 9 | Cryo-EM density map validation of CarD-DNA interactions and σ factor conformations in mycobacterial transcription complexes (related to Figure 4).

A, D. Composite views of CarD and the σ2 domain interacting with DNA in the *Mtb* IC6 **(A)** and *Mtb* PIC7 **(D)**. CarD (orange cartoon), the σ2 domain (magenta cartoon), and DNA strands (template: cyan; non-template: warm pink) are shown as molecular models overlaid on cryo-EM density maps (threshold = 0.19, 50% transparency).

B, E. Regions of CarD-DNA interactions (dashed boxes) are highlighted by a dashed frame and are enlarged on the right. CarD binds the template strand in *Mtb* IC6 **(B)**, while it engages the non-template strand in *Mtb* PIC7 **(E)**. Critical residues mediating these interactions are depicted as stick models with residue labels.

C, F. σ factor conformations in *Mtb* IC6 **(C)** and PIC7 **(F)** are shown as cartoon models superimposed on cryo-EM density maps (threshold = 0.19, 50% transparency).

Supplementary Tables

Supplementary Table 1. Reported RNAP–nucleic acid scaffold designs in Mycobacterium tuberculosis and Mycobacterium smegmatis

System / factor	Complex type	Scaffold design	Bubble length (nt)	Bubble/Hybrid density	Published
Mtb/σ^{E}, σ^{H}	Initiation	Pre-melted	12 nt (mismatch)	resolved	(2019) Nucleic Acids Res **47**: 7094-7104
Mtb/σ^{H}	Initiation	Pre-melted	12 nt (mismatch)	resolved	(2019) Nat Commun **10**: 1-14
Mtb/σ^{L}	Initiation	Pre-melted	7 nt (mismatch)	resolved	(2019) Nat Commun **10**: 710-710
Mtb/σ^{A}, σ^{H}	Initiation	Pre-melted	2-10 nt (mismatch)	resolved	(2020) PNAS **117**: 5801-5809
Mtb/σ^{A}, GlnR	Initiation	Pre-melted	13 nt (mismatch)	resolved	(2023) PNAS **120**: e2300282120
Mtb/σ^{A}	Initiation	Pre-melted	8 nt (mismatch)	resolved	(2017) Mol Cell **66**: 169-179.e8
Mtb/PhoP, σ^{A}	Initiation	Pre-melted	13 nt (mismatch)	resolved	(2025) Nat Commun **16**: 1573-1573
Msm/PafBC, σ^{A}	Initiation	Pre-melted	13 nt (mismatch)	resolved	(2021) Sci Adv **7**: eabl4064
Msm/RbpA , σ^{A}	Initiation	Pre-melted	13 nt (mismatch)	resolved	(2017) Nat Commun **8**: 16072-16072
Msm/HelD, σ^{A}	Initiation	Pre-melted	13 nt (mismatch)	missing	(2024) Nat Commun **15**: 8740-8740
Mtb/NusG	Elongation	Pre-melted	10 nt (mismatch)	resolved	(2023) PNAS **120**: e2218516120
Mtb/NusG	Elongation	Pre-melted	9 nt (mismatch)	resolved	(2023) Mol Cell **83**: 1474-1488
Mtb/σ^{A}	RPo	non	13 nt match	partially missing	(2019) Nature **565**: 382-385
Mtb, Msm/σ^{A}	RPo	non	13 nt match	partially missing	(2020) PNAS **117**: 30423-30432
Mtb/WhiB7, σ^{A}	RPo	non	match	missing	(2021) Mol Cell **81**: 2875-2886
Mtb/RbpA, σ^{A}	RPo	non	match	missing	(2018) Elife **7**: e34823
Msm/RbpA, σ^{A}	RPo	non	match	missing	(2018) Nat Commun **9**: 4147-4147
Msm/RbpA , σ^{A}	RPo	non	match	missing	(2017) Elife **6**: e22520

Supplementary Table 2. Cryo-EM data statistics (IC6, IC7 and PIC7).

	Mtb IC6 (EMD-63729) (PDB 9M98)	*Mtb* IC7 (EMD-63730) (PDB 9M9D)	*Mtb* PIC7 (EMD-63731) (PDB 9M9E)
**Data collection and processing**			
Magnification	81000	81000	81000
Voltage (kV)	300	300	300
Electron exposure (e–/Å2)	55	55	55
Defocus range (μm)	-1.0 to -1.8	-1.0 to -1.8	-1.0 to -1.8
Pixel size (Å)	0.89	0.89	0.89
Symmetry imposed	C1	C1	C1
Initial particle images (no.)	369,460	1300,971	1300,971
Final particle images (no.)	65,079	75,465	65,908
Map resolution (Å)	3.5	3.3	3.3
FSC threshold	0.143	0.143	0.143
Map resolution range (Å)	2.5 – 8.5	2.5 – 8.5	2.5 – 8.5
**Refinement**			
Initial model used (PDB code)	5ZX2	5ZX2	5ZX2
Model resolution (Å)	3.5	3.3	3.3
FSC threshold	0.143	0.143	0.143
Model resolution range (Å)	∞ to 3.33	∞ to 3.33	∞ to 3.21
Map sharpening B factor (Å2)	-114.1	-117.7	-111.1
Model composition			
Non-hydrogen atoms	26,876	26,698	26,827
Protein residues	3232	3224	3234
Ligands	3	3	3
B factors (Å2)			
Protein	161.06	154.8	151.07
Ligand	203.47	155.5	156.56
R.m.s. deviations			
Bond lengths (Å)	0.005	0.004	0.004
Bond angles (°)	0.640	0.679	0.609
Validation			
MolProbity score	1.90	1.93	1.75
Clashscore	11.52	13.19	9.41
Poor rotamers (%)	0	0	0.04
Ramachandran plot			
Favored (%)	95.39	95.65	96.27
Allowed (%)	4.58	4.19	3.64
Disallowed (%)	0.03	0.16	0.09

Supplementary Table 3. Cryo-EM data statistics(N-IC6, N-IC7 and N-PIC7).

	Mtb N- IC6<br>EMD-66363	*Mtb* N-IC7<br>EMD-66364	*Mtb* N-PIC7<br>EMD-66362
**Data collection and processing**			
Magnification	81000	81000	81000
Voltage (kV)	300	300	300
Electron exposure (e–/Å2)	55	55	55
Defocus range (μm)	-1.0 to -1.8	-1.0 to -1.8	-1.0 to -1.8
Pixel size (Å)	0.87	0.87	0.87
Symmetry imposed	C1	C1	C1
Initial particle images (no.)	222,146	332,402	332,402
Final particle images (no.)	25,048	15,872	31,912
Map resolution (Å)	3.6	4.0	3.9
FSC threshold	0.143	0.143	0.143
Map resolution range (Å)	2.5 – 8.5	2.5 – 8.5	2.5 – 8.5

Supplementary Table 4. RNAP and σ^E domain and structural modules.

Domain/Module	Subunit	Residues
Core module	α1	all
	α2	all
	ω	all
	β	28-53, 437-747,879-1036
	β'	418-443, 579-863
Swivel module	β	1067-1150
	β’	3-417, 444-495, 864-1011, 1026-1281
Flap-tip helix	β	815-828
C-terminal helix	β	1096-1106
Coiled-coil	β’	341-392
Rudder	β’	393-400
Lid	β’	323-340
Bridge helix (BH)	β’	846-884
SI	β’	142-227
Jaw	β’	1041-1115
Gate loop	β	280-291
Protrusion	β	54-176, 381-444
Dock	β’	444-495
Shelf	β’	864-1011, 1026-1040, 1116-1218
Lobe	β	177-379
NADFDGD Loop	β’	533-539
Clamp	β	1117-1150
	β’	3-413,1219-1245
σ^E	σ2	80-155
	σ3.2	156-183
	σ4	184-242
CarD	α3	85-98
	α5	121-143

Supplementary Table 5. The plasmids and strains used in this study.

Plasmids	Strains	Sources
pETDuet-*rpoB*-*rpoC* (encodes *Mtb* β and His10-β')	BL21 (DE3)	this study
pRSFDuet-*rpoA*-*rpoZ* (encodes *Mtb* α and ω)	BL21 (DE3)	this study
pETDuet-σ^E (encodes *Mtb* His6-σ^E)	BL21 (DE3)	this study
pRSFDuet-CarD (encodes *Mtb* His6-CarD)	BL21 (DE3)	this study
pETDuet-σ^E-R226A (encodes *Mtb* His6-σ^E-R226A)	BL21 (DE3)	this study
pETDuet-σ^E-R228A (encodes *Mtb* His6-σ^E-R226A)	BL21 (DE3)	this study
pETDuet-σ^E -R231A (encodes *Mtb* His6-σ^E-R231A)	BL21 (DE3)	this study
pRSFDuet-CarD-W85A (encodes *Mtb* His6-CarD-W85A)	BL21 (DE3)	this study
pRSFDuet-CarD-Y89A (encodes *Mtb* His6-CarD-Y89A)	BL21 (DE3)	this study
pRSFDuet-CarD-R126A (encodes *Mtb* His6-CarD-R126A)	BL21 (DE3)	this study

Supplementary Table 6. Primers and sequences used in this study.

Primer name	Sequence (5'-3')
rpoB-F	gggaattccatatgttggcagattcccgccagagcaaaacagcc
rpoB-R	ccgctcgagttacgcaagatcctcgacacttgcggattcgtt
rpoC-F	catgccatggtgctcgacgtcaacttcttcgatgaactcc
rpoC-R	cgggatccttaatgatgatgatgatgatgatgatgatgatg gcggtagtcgctgtagccgtagtcgtccag
rpoA-F	gggaattccatatgctgatctcacagcgccccaccc
rpoA-R	ccgctcgagttaaagctgttcggtttcggcgtagtcc
rpoZ-F	catgccatggtgagtatctcgcagtccgacgcgt
rpoZ-R	cgggatccttactcgccctcggtgtgctcgagcaga
σ^E-F	gggaattccatatgcaccaccaccaccaccac atggaactcctcggcggaccccggg
σ^E-R	ccgctcgagttagcgaactgggttgacgtgaactgcg
CarD-F	catgccatggcccaccaccaccaccaccac atgattttcaaggtcggagacaccgt
CarD-R	cgggatccttaagacgcggcggctaaaacctcgtca
σ^E-R226A-F	aagctcgggacggtaGCCagccggatacaccgc
σ^E-R226A-R	gcggtgtatccggctGGCtaccgtcccgagctt
σ^E-R228A-F	gggacggtacgtagcGCCatacaccgcggacgc
σ^E-R228A-R	gcgtccgcggtgtatGGCgctacgtaccgtccc
σ^E-R231A-F	cgtagccggatacacGCCggacgccaggcactg
σ^E-R231A-R	cagtgcctggcgtccGGCgtgtatccggctacg
CarD-W85A-F	gaggagccgacgaacGCCtcacgtcgttacaag
CarD-W85A-R	cttgtaacgacgtgaGGCgttcgtcggctcctc
CarD-Y89A-F	aactggtcacgtcgtGCCaaggcgaacctcgag
CarD-Y89A-R	ctcgaggttcgccttGGCacgacgtgaccagtt
CarD-R126A-F	tcggccggtgagaagGCCatgctggccaaggcc
CarD-R126A-R	ggccttggccagcatGGCcttctcaccggccga

digestion site: red; his-label: blue; mutation site: uppercase;

Supplementary Table 7. Promoter DNA scaffolds used for cryo-EM sample preparation, FRET assay for CarD-dependent RPo stabilization, and in vitro transcription assays.

Promoter	DNA strand (5'-3')
σ^E-pre-melt-NT	CGGAACTTTTAGTGCTAATTATTGTCAAAACCATTGTCACGGATGCAG
σ^E-pre-melt-T	CTGCATCCGTGAGTCGAGGGTGTTCAATAATTAGCACTAAAAGTTCCG
psigB-*Mango*	CTCAGGACTTTCTCAGGTCTTCGGCAGATTCCTGCACGTCACAGGGCGT CAGATCACTGCTGGGTGGGAACTCAAAGTCCGGCTTTGTCGTTAAACCC CATGGGCATTTCGAACGGACCCCGAATGGAGGTGTCGTGGACTCGTTTA ACCCGGGCACGTACGAAGGAAGGATTGGTATGTGGTATATTCGTACGTG CCGGCCTGCTGGTAATCGCAggccttttttatttaagggcag
σ^E-NT-BHQ	CGGAACTTTTAGTGCTAATTATGGTTGTACCTCGACTCACGGATGCAG
σ^E--T-CY3	CTGCATCCGTGAGTCGAGGTACAACCATAATTAGCACTAAAAGTTCCG
σ^E-match-NT	CGGAACTTTTAGTGCTAATTATGGTTGTACCTCGACTCACGGATGCAG
σ^E-match-T	CTGCATCCGTGAGTCGAGGTACAACCATAATTAGCACTAAAAGTTCCG
Match-RNA6	ACUCAC
Match-RNA7	ACUCACG

-10 element: magenta; -35 element: blue; start site: red; mango sequences: green; terminator sequences: lowercase; BHQ label: purple; Cy3 label: yellow.
